# Secreted Toxins From *Staphylococcus aureus* Strains Isolated From Keratinocyte Skin Cancers Mediate Pro-tumorigenic Inflammatory Responses in the Skin

**DOI:** 10.3389/fmicb.2021.789042

**Published:** 2022-01-25

**Authors:** Annika Krueger, Julian Zaugg, Sarah Chisholm, Richard Linedale, Nancy Lachner, Siok Min Teoh, Zewen K. Tuong, Samuel W. Lukowski, Mark Morrison, H. Peter Soyer, Philip Hugenholtz, Michelle M. Hill, Ian H. Frazer

**Affiliations:** ^1^Faculty of Medicine, The University of Queensland Diamantina Institute, Translational Research Institute, The University of Queensland, Woolloongabba, QLD, Australia; ^2^Australian Centre for Ecogenomics, School of Chemistry and Molecular Biosciences, The University of Queensland, St Lucia, QLD, Australia; ^3^Department of Medicine, University of Cambridge School of Clinical Medicine, Cambridge, United Kingdom; ^4^Cellular Genetics, Wellcome Sanger Institute, Hinxton, United Kingdom; ^5^The Institute for Molecular Bioscience, The University of Queensland, St Lucia, QLD, Australia; ^6^Dermatology Research Centre, The University of Queensland Diamantina Institute, The University of Queensland, Brisbane, QLD, Australia; ^7^Dermatology Department, Princess Alexandra Hospital, Brisbane, QLD, Australia; ^8^QIMR Berghofer Medical Research Institute, Brisbane, QLD, Australia; ^9^The University of Queensland Centre for Clinical Research, Faculty of Medicine, The University of Queensland, Brisbane, QLD, Australia

**Keywords:** skin microbiota, microbiome, squamous cell carcinoma, actinic keratosis, *S. aureus*, proteomics, multi-omics, inflammation

## Abstract

Squamous cell carcinoma (SCC) is a common type of skin cancer that typically arises from premalignant precursor lesions named actinic keratoses (AK). Chronic inflammation is a well-known promoter of skin cancer progression. AK and SCC have been associated with an overabundance of the bacterium *Staphylococcus aureus* (*S. aureus*). Certain secreted products from *S. aureus* are known to promote cutaneous pro-inflammatory responses; however, not all *S. aureus* strains produce these. As inflammation plays a key role in SCC development, we investigated the pro-inflammatory potential and toxin secretion profiles of skin-cancer associated *S. aureus*. Sterile culture supernatants (“secretomes”) of *S. aureus* clinical strains isolated from AK and SCC were applied to human keratinocytes *in vitro.* Some *S. aureus* secretomes induced keratinocytes to overexpress inflammatory mediators that have been linked to skin carcinogenesis, including IL-6, IL-8, and TNFα. A large phenotypic variation between the tested clinical strains was observed. Strains that are highly pro-inflammatory *in vitro* also caused more pronounced skin inflammation in mice. Proteomic characterization of *S. aureus* secretomes using mass spectrometry established that specific *S. aureus* enzymes and cytolytic toxins, including hemolysins, phenol-soluble modulins, and serine proteases, as well as currently uncharacterized proteins, correlate with the pro-inflammatory *S. aureus* phenotype. This study is the first to describe the toxin secretion profiles of AK and SCC-associated *S. aureus*, and their potential to induce a pro-inflammatory environment in the skin. Further studies are needed to establish whether these *S. aureus* products promote SCC development by mediating chronic inflammation.

## Introduction

Actinic keratoses (AK) are pre-malignant skin lesions that, over time, can progress to intraepidermal carcinoma (IEC) and invasive squamous cell carcinoma (SCC) (Brantsch et al., 2008; Soyer et al., 2012). AK formation on photo-damaged skin is common in fair-skinned individuals and its incidence rises substantially with age (Soyer et al., 2012). Exposure to UV radiation is the primary risk factor driving the progression from AK to SCC. Chronic inflammation is also recognized as a key driver for the malignant transformation of photo-damaged skin (Mueller, 2006; Grivennikov et al., 2010). IEC and SCC more frequently develop in chronically inflamed skin sites, such as burns, wounds, and ulcers, and are common in individuals with inflammatory skin conditions (Mueller, 2006). Inflammation predisposes to genomic instability due to inflammation-linked production of reactive oxygen species which can cause DNA damage (Mantovani et al., 2008). Additionally, inflammation-associated secretion of soluble inflammatory mediators and growth factors, such as IL-6, IL-8, and TNF-α, promote cell proliferation, inhibit apoptosis, and activate survival pathways (Lin and Karin, 2007; Grivennikov et al., 2010). Inflammatory mediators recruit leukocytes to the tumor site where these trigger stroma remodeling and angiogenesis, which, in turn, supports the high demand for nutrients and oxygen of fast proliferating tumor cells (Grivennikov et al., 2010).

Human skin is colonized by bacteria, archaea, fungi, and viruses, and the skin microbiome is known to influence the inflammatory state of the skin (Prescott et al., 2017; Byrd et al., 2018). Hence, it is plausible that cutaneous microbiota impact the malignant transformation of skin epithelial cells by mediating lasting pro-inflammatory signaling. However, relatively little is known about the impact of the local skin microbiota on the development of cutaneous SCC. Several studies have reported skin microbiome changes occurring in AK and SCC, when compared to non-malignant skin, with an overabundance of the Gram-positive bacterium *Staphylococcus aureus* (*S. aureus*) in AK and SCC being the most prominent finding (Kullander et al., 2009; Wood et al., 2018; Andersson et al., 2019; Madhusudhan et al., 2020).

*S. aureus* has the capacity to produce toxins and superantigens that can mediate pronounced cutaneous pro-inflammatory immune responses (Otto, 2014; Liu et al., 2017, 2018). However, this species is also well-known for its wide phenotypic heterogeneity and diverse virulence potential, which is largely mediated *via* its highly variable exoproteome (Ziebandt et al., 2010; Bonar et al., 2016). Thus, the quality and quantity of secreted bacterial products (the “secretome”) of *S. aureus* predominately determines the host response and distinct clinical manifestations. To our knowledge, the expression profiles of skin-cancer associated clinical *S. aureus* strains, and their potential to induce pro-inflammatory responses in the skin, have previously not been investigated. Here we demonstrate that secreted products from AK, IEC, and SCC-associated *S. aureus* can trigger keratinocytes to produce inflammatory cytokines that are typically upregulated in SCC. The potency of these clinical *S. aureus* strains to induce pro-tumorigenic inflammatory responses varied widely, due to differences in secretome composition. Our study indicates that certain *S. aureus* strains and products may mediate inflammation in premalignant skin lesions.

## Results

### Genetic Characterization of *Staphylococcus aureus* Isolates From Subjects With Squamous Skin Cancer

*S. aureus* overabundance is associated with premalignant skin and skin cancers (Kullander et al., 2009; Wood et al., 2018; Andersson et al., 2019; Madhusudhan et al., 2020). To assess the characteristics of *S. aureus* relevant and specific to the different progression stages of SCC, we established a biobank of 67 *S. aureus* isolates from non-malignant photo-damaged skin (*n* = 13), AK (*n* = 8), IEC (*n* = 8), or SCC (*n* = 38) from 16 subjects (Supplementary Table 1). For some subjects, multiple *S. aureus* cultures from the same skin swab were isolated to determine whether these lesions were typically populated by the same strain or a mix of genetically distinct *S. aureus*. Draft genome sequences were derived from 34 of our clinical isolates. Multilocus sequence typing and phylogenetic analysis of these isolates showed that *S. aureus* recovered from the same skin site were closely related, and that *S. aureus* isolated from the same subject but from different skin sites were also genetically similar (Supplementary Table 1 and Supplementary Figure 1). As strains isolated from the same skin site were genetically and phenotypically alike, we only present data for one representative isolate per skin site in the subsequent functional studies.

### *Staphylococcus aureus* Secretions Trigger Keratinocytes to Produce Cytokines Overexpressed in Squamous Skin Cancer

**FIGURE 1:**
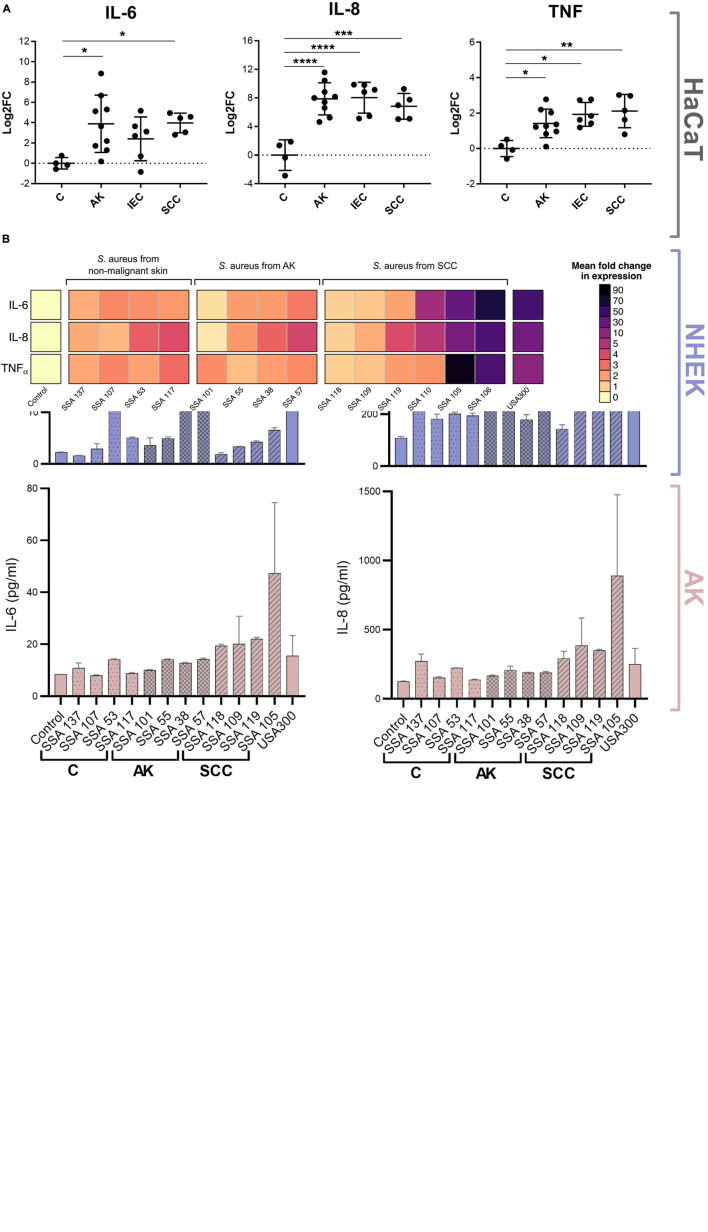
*Staphylococcus aureus* products induce transcription of cytokines that are commonly overexpressed in AK, IEC, and SCC in human keratinocytes. **(A)** Biopsies of actinic keratoses (AK; *n* = 9), intraepithelial cancers (IEC; *n* = 6), and squamous cell carcinomas (SCC; *n* = 5) assayed for IL-6, IL-8, and TNFα mRNA. Results (mean ± 1 SD) are normalized to results from healthy skin (C; *n* = 4). *p ≤ 0.05, **p ≤ 0.01, ***p ≤ 0.001, and ****p ≤ 0.0001; Kruskal-Wallis test. **(B)** Expression of cytokine mRNAs by HaCaT keratinocytes exposed for 6 h to secretome from *S. aureus* isolated from control skin, AK or SCC lesions, normalized to mRNA expression induced by the control defined *Staphylococcus* media (DSM). Mean of three biological replicates ± 1 SD shown. **(C)** HaCaT keratinocytes and primary human keratinocytes originating from foreskin (NHEK) and actinic keratosis lesions (AK) were exposed to secretome from *S. aureus* strains isolated from non-malignant skin (C), AK, and SCC, or uninoculated DSM as control. After 24 h, cytokine levels in keratinocyte conditioned media were measured *via* multiplex bead-based cytometric assay. Displayed is the mean of two biological replicates ± 1 SD.

The pro-inflammatory cytokines IL-6, IL-8, and TNFα possess tumor-promoting functions and increased tissue levels are associated with many types of cancer (Gambichler et al., 2006; Lin and Karin, 2007; Lederle et al., 2011; Moussai et al., 2011; Ghahartars et al., 2021). Consistent with this observation, we found these cytokines were overexpressed in AK, IEC, and SCC biopsies when compared to non-malignant skin (Figure 1A). To determine if *S. aureus* secreted products can directly contribute to this overexpression of pro-tumorigenic cytokines in epithelial cells, we first measured by qPCR the level of expression of IL-6, IL-8, and TNFα genes in human HaCaT keratinocytes, before and after exposure to filter-sterilized culture supernatants from *S. aureus* isolated from non-malignant skin (*n* = 4), AK (*n* = 4), and SCC (*n* = 6). IL-6, IL-8, and TNFα transcripts were generally increased in keratinocytes challenged with *S. aureus* secretomes when compared to keratinocytes exposed to *Staphylococcus* medium only, with certain secretomes being particularly potent inducers (Figure 1B). The genes encoding each of the three cytokines were induced to a similar extent by any given *S. aureus* secretome. The gene expression levels by qPCR were mirrored for IL-6 and IL-8 by comparable increases in protein secretion measured using a cytometric bead array (Figure 1C). Further, the levels of secreted IL-6 and IL-8 protein in response to *S. aureus* secretomes from HaCaT keratinocytes was comparable to those observed for primary human keratinocytes, isolated from normal skin and actinic keratosis (Figure 1C).

### Large Heterogeneity of *Staphylococcus aureus* Clinical Isolates to Promote IL-6 Secretion in Human Keratinocytes

**FIGURE 2:**
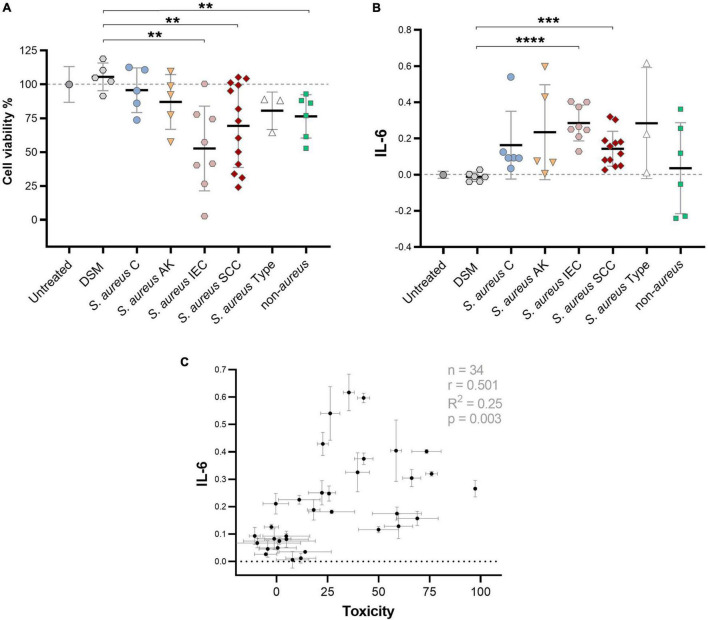
Large variance of clinical *S. aureus* to induce cytotoxic and IL-6 responses in keratinocytes. **(A)** Percent viability of HaCaT keratinocytes after challenge with secretome from *S. aureus* isolates obtained from control skin (C), AK, IEC, and SCC, *S. aureus* lab type strains USA300, ATCC25923, and ATCC29213, as well as secretome from other *Staphylococcus* species (*S. epidermidis* and *S. capitis* isolated from AK, IEC, or SCC) as measured *via* MTT assay. The different batches of the bacterial media control (DSM) used to culture the tested staphylococci strains served as negative control. Normalized results are displayed as percentage of viable cells (untreated = 100% viable; cells killed with lysis buffer = 0%). Bars indicate the group mean ± 1 SD; each data point represents the mean of triplicate measurement of a different isolate; DSM *n* = 3; untreated control *n* = 12. Statistical significance between DSM and treatment groups determined by unpaired *t*-test; two-tailed *p*-value indicated. **(B)** Relative IL-6 levels as measured by colorimetric cell reporter assay (absorbance at 650 nm) in keratinocyte conditioned media after exposure to secretome from *S. aureus* or other *Staphylococcus* species (untreated cells = baseline). *S. aureus* secretome samples are grouped based on swab origin. One data point represents IL-6 activity of a single isolate secretome (mean of triplicate measurements). Bars indicate mean ± 1 SD. Statistical significance between DSM and treatment groups determined by unpaired *t*-test; two-tailed *p*-value indicated. **(C)** Pearson’s correlation analysis between keratinocyte IL-6 levels and toxicity in response to treatment with 34 *S. aureus* secretome samples (two-tailed *p*-value 0.0025). **p ≤ 0.01, ***p ≤ 0.001, and ****p ≤ 0.0001.

As the induction of pro-tumorigenic cytokines from keratinocytes by *S. aureus* secretomes appeared highly strain-specific, we next investigated the potency of a larger number of secretomes from different clinical isolates, originating from non-malignant control (C) (*n* = 5); AK (*n* = 5); IEC (*n* = 8); SCC (*n* = 13), to induce IL-6 secretion from human keratinocytes by using a reporter cell assay. Additionally, keratinocyte viability after exposure to secretomes was assessed, to measure toxicity-dependent effects. *S. aureus* secretomes varied in their ability to reduce cell viability (Figure 2A) and induce the secretion of IL-6 (Figure 2B) in HaCaT keratinocytes. Cytotoxic *S. aureus* secretomes tended to induce higher levels of IL-6. However, many secretome samples were non-toxic, but yet triggered IL-6 secretion, indicating that cytokine induction was not directly linked to toxicity (Figure 2C). A significant increase in keratinocyte IL-6 secretion was observed in response to secretomes from *S. aureus* originating from IEC (*p* = 0.0001) and SCC (*p* = 0.0001), when compared to the media control (Figure 2B). Notably, secretomes from other *Staphylococcus* species isolated from AK, IEC, and SCC swabs, including *S. epidermidis* and *S. capitis*, induced lower levels of IL-6 than lesion-associated *S. aureus* secretomes (Figure 2B). The keratinocyte IL-6 secretion mediated by *S. aureus* supernatant was not dependent on bacterial culture conditions, or on bacterial cell counts at the time of supernatant collection, and was comparable to IL-6 secretion in response to keratinocyte infection with unfiltered *S. aureus* supernatant containing live cells (Supplementary Figure 2). To validate the IL-6 results obtained using a cell-based reporter assay, a cytometric bead array was used as an additional method to measure secreted IL-6 levels from keratinocytes after challenge with 12 different *S. aureus* secretomes. The IL-6 concentration quantified *via* bead array closely matched the IL-6 concentration measured *via* cell reporter assay, confirming reliability of the IL-6 results (R^2^ = 0.97; *p* < 0.0001) (Supplementary Figure 3).

### IL-6-Inducing *Staphylococcus aureus* Stimulate More Pronounced Pro-inflammatory Skin Responses in Mice

**FIGURE 3:**
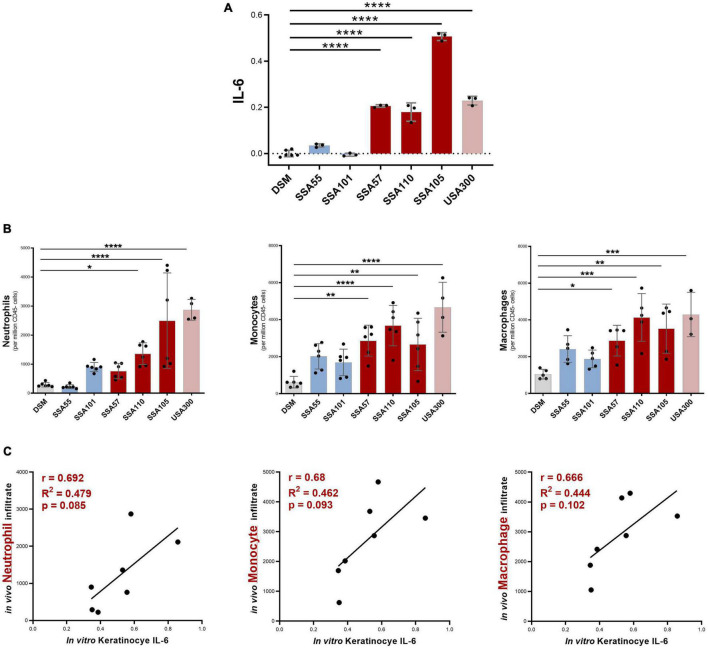
*Staphylococcus aureus* secretome-induced IL-6 levels from keratinocyte *in vitro* predict neutrophil, monocyte, and macrophage recruitment in response to secretome *in vivo*. **(A)** Six *S. aureus* secretomes were selected for *in vivo* studies which had no significant effect (blue), or a strong positive effect (red), on keratinocyte IL-6 secretion *in vitro* (relative IL-6 concentration based on OD value from cell reporter assay; values normalized to baseline = DSM; mean ± 1 SD: *n* = 3 per secretome; Dunnett’s multiple comparisons test ****P < 0.0001). **(B)** Immune infiltrate in mouse ear skin 24 h post injection with *S. aureus* secretomes, determined by flow cytometry (mean ± 1 SD: *n* = 6 per secretome); Statistical significance determined by Dunnett’s multiple comparisons test. **(C)** Correlation between *in vitro* keratinocyte IL-6 induction and *in vivo* neutrophil, monocyte, and macrophage infiltrate in response to *S. aureus* secretome exposure. *p ≤ 0.05, **p ≤ 0.01, ***p ≤ 0.001 and ****p ≤ 0.0001.

IL-6 trans-signaling typically induces local and systemic pro-inflammatory responses, in part by recruiting and activating innate immune cells (Turner et al., 2014). To test whether *S. aureus* secretomes that promoted a high expression of IL-6 in keratinocytes *in vitro* were also potent activators of innate immune responses in the skin *in vivo*, a mouse model of intradermal injection of *S. aureus* secretome into the ear skin was utilized. Mouse ears were injected either with secretome that had no significant effect on IL-6 production in cultured keratinocytes, or secretome that was highly IL-6-inducing (Figure 3A). At 24 h post-injection, most IL-6-inducing secretomes caused a statistically significant increase in neutrophil, monocyte, and macrophage infiltrates, compared to ears injected with the bacterial media control (Figure 3B). *S. aureus* secretomes that had no effect on keratinocyte IL-6 production *in vitro* produced non-significant increases in immune populations in the murine skin (Figure 3B). A substantial positive correlation between the *in vitro* IL-6 keratinocyte signal and the *in vivo* infiltrates was observed, though not statistically significant (Figure 3C).

### A Specific Secretome Profile Is Linked to the Pro-inflammatory *Staphylococcus aureus* Phenotype

To identify bacterial proteins contributing to the cytokine inducing *S. aureus* phenotype, the protein content of the secretome from 28 clinical isolates derived from non-malignant photo-damaged skin (*n* = 10); AK (*n* = 8); SCC (*n* = 10), and three *S. aureus* type strains, was characterized by shotgun mass spectrometry (MS) following trypsin digest. The IL-6 levels secreted by keratinocytes in response to these MS-characterized secretomes was used as representative readouts for inflammatory potential, and ranged from non-inflammatory to highly pro-inflammatory secretomes (Supplementary Table 2). A total of 348 *S. aureus* proteins were identified across the 31 secretomes and the relative quantity of these proteins in each secretome sample was determined by MaxQuant label-free quantification. Using Spearman’s correlation with an adjusted *p* < 0.05 as cut off, we identified 37 *S. aureus* proteins positively correlated, and three negatively correlated, with IL-6 induction (Table 1). IL-6-inducing potential of *S. aureus* secretomes was correlated with levels of potent cytolytic toxins including leucocidins, hemolysins, and phenol-soluble modulins (PSMs), as well as the SarA protein, which is involved in the regulation of these toxins. Additionally, multiple serine proteases and cell wall modifying enzymes such as lipoteichoic acid synthase were associated with the ability of *S. aureus* to cause pro-inflammatory skin responses. Interestingly, we also identified six as-yet uncharacterized proteins associated with the pro-inflammatory *S. aureus* phenotype, including a probable CtpA-like serine protease, a putative phosphoesterase, and an uncharacterized peptidase and lipoprotein.

**TABLE 1:** *S. aureus* peptides and proteins that are significantly correlated with *S. aureus*-mediated IL-6 induction in human keratinocytes.

Gene	Protein	protID	r	adj.p
*sarA*	Transcriptional regulator SarA	Q7A732	0.76	0.00032
*hla*	Alpha-hemolysin	Q2G1X0	0.73	0.00066
*lip1*	Lipase 1	Q8NUI5	0.70	0.0018
*sucD*	Succinate-CoA ligase subunit alpha	Q8NX01	0.66	0.0046
*SAV1420*	Probable CtpA-like serine protease	Q99U67	0.66	0.0046
*NWMN_1872*	65 kDa membrane protein	A6QIG2	0.64	0.0053
*atl*	Bifunctional autolysin	Q6GAG0	0.64	0.0056
*atpF*	ATP synthase subunit b	Q99SF1	0.64	0.0056
*spsB*	Signal peptidase IB	Q6GIC3	0.63	0.0070
*rplE*	50S ribosomal protein L5	Q99S33	0.61	0.0088
*ltaS*	Lipoteichoic acid synthase	Q99VQ4	0.61	0.0094
*splA*	Serine protease SplA	Q99T60	0.60	0.011
*lip2*	Lipase 2	Q8NYC2	0.60	0.011
*adk*	Adenylate kinase	Q6GEK4	0.58	0.014
*fda*	Fructose-bisphosphate aldolase class 1	Q5HCU6	0.58	0.014
*rpsH*	30S ribosomal protein S8	Q6GEJ7	0.58	0.014
*SAV1015*	Putative phosphoesterase SAV1015	Q99V77	0.58	0.014
*srrA*	Transcriptional regulatory protein SrrA	Q9L524	0.57	0.016
*efp*	Elongation factor P	Q6GGH0	0.56	0.019
*lukEv*	Leucotoxin LukEv	Q2FXB0	0.55	0.020
*rpmF*	50S ribosomal protein L32	Q6GHV8	0.55	0.020
*scdA*	Iron-sulfur cluster repair protein ScdA	Q8NYH4	0.55	0.023
*SAV1708*	Uncharacterized peptidase SAV1708	Q99TF5	0.54	0.023
*hlgA*	Gamma-hemolysin component A	Q6G6Q2	0.54	0.023
*SACOL0486*	Uncharacterized lipoprotein SACOL0486	Q5HIN1	0.54	0.024
*deoC2*	Deoxyribose-phosphate aldolase 2	Q8NVF5	0.54	0.025
*qoxA*	Probable quinol oxidase subunit 2	Q99V36	0.54	0.025
*folD*	Bifunctional protein FolD	Q99V34	0.53	0.028
*psmA4*	Phenol-soluble modulin α4 peptide	P0C827	0.52	0.032
*sodA*	Superoxide dismutase [Mn/Fe] 1	Q6GGE6	0.51	0.039
*splC*	Serine protease SplC	Q7A4Y2	0.50	0.040
*psmA3*	Phenol-soluble modulin α3 peptide	P0C814	0.50	0.040
*ndk*	Nucleoside diphosphate kinase	Q6GGU2	0.50	0.040
*clpL*	Clp protease ATP-binding subunit ClpL	Q99R88	0.50	0.040
*tsf*	Elongation factor Ts	Q8NWZ6	0.49	0.049
*SAS1797*	Uncharacterized protein SAS1797	Q6G859	0.48	0.050
*upp*	Uracil phosphoribosyltransferase	Q6GEW3	0.48	0.050
*isdB*	Iron-regulated surface determinant protein B	Q99UX5	−0.61	0.0094
*guaB*	Inosine-5′-monophosphate dehydrogenase	Q8NY70	−0.58	0.014
*lytN*	Probable cell wall hydrolase LytN	Q9ZNI1	−0.48	0.050

*Listed are S. aureus proteins which relative amount within sterile bacterial supernatant (determined via mass spectrometry label-free quantification) significantly correlated to the secretomes’ ability to induce IL-6 in keratinocytes as determined by Spearman’s correlation. Test threshold was set at an FDR of 5%. Separated on the bottom of the table are proteins with negative correlation. adj.p, Adjusted p-value; r, coefficient of correlation.*

**FIGURE 4:**
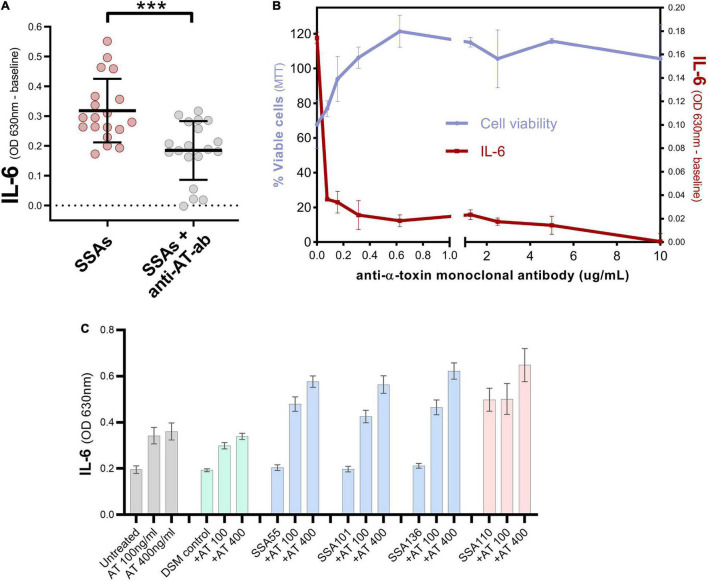
α-toxin contributes to the induction of IL-6 by *S. aureus* secretomes. **(A)** Keratinocyte secreted IL-6 levels after exposure to 19 different *S. aureus* secretomes with or without addition of anti-α-toxin polyclonal antibody (mean ± 1 SD: *n* = 3 per secretome). IL-6 absorbance values were normalized to baseline i.e., IL-6 value for untreated cells. Unpaired *t*-test; two-tailed ***p = 0.0003. **(B)** Validation of polyclonal antibody data, by testing different concentrations of anti-α-toxin monoclonal antibody in combination with secretome from a SCC-derived *S. aureus* isolate SSA105 known to contain high levels of α-toxin. Percentage of viable keratinocytes (left axis) and keratinocyte IL-6 response (IL-6 absorbance values baseline-corrected to untreated cells, right axis) after exposure to secretome from SSA105 ± anti-α-toxin monoclonal antibody. Neutralizing α-toxin results in loss of cytotoxic action of the tested *S. aureus* secretome and diminishes its ability to stimulate IL-6 secretion from HaCaT cells. Error bars indicate mean and standard deviation (*n* = 2). **(C)** Keratinocyte secreted IL-6 levels after exposure to *S. aureus* secretomes with or without addition of 100 or 400 ng/ml recombinant α-toxin (mean ± 1 SD: *n* = 3 per secretome), compared to untreated cells (gray) and control cells treated with uninoculated bacterial media DSM (green). Secretome from *S. aureus* isolates SSA55, SSA55, and SSA55 naturally contain very low levels of α-toxin (blue), whereas SSA110 naturally produces high levels of α-toxin (red).

Neutralization of one of the most positively correlated proteins, α-toxin, caused a significant reduction in the ability of secretomes to induce IL-6 in cultured keratinocytes (Figures 4A,B). Conversely, the addition of recombinant α-toxin to *S. aureus* secretomes that typically had no impact on keratinocyte IL-6 levels caused these secretomes to become potent activators of IL-6 secretion (Figure 4C).

Some *S. aureus* strains may generally secrete higher levels of bacterial proteins than others, which could affect keratinocyte responses. Indeed, the keratinocyte IL-6 readout also showed a significantly positive correlation to the total protein content of *S. aureus* secretomes (Supplementary Figure 4A). However, this was not a culturing-dependent artifact caused by differences in growth since the overall protein content of *S. aureus* secretome did not correlate with bacterial cell density at time of harvest (Supplementary Figure 4B).

### Pro-inflammatory *Staphylococcus aureus* Secretome Regulation Is Independent of Genotype

**FIGURE 5:**
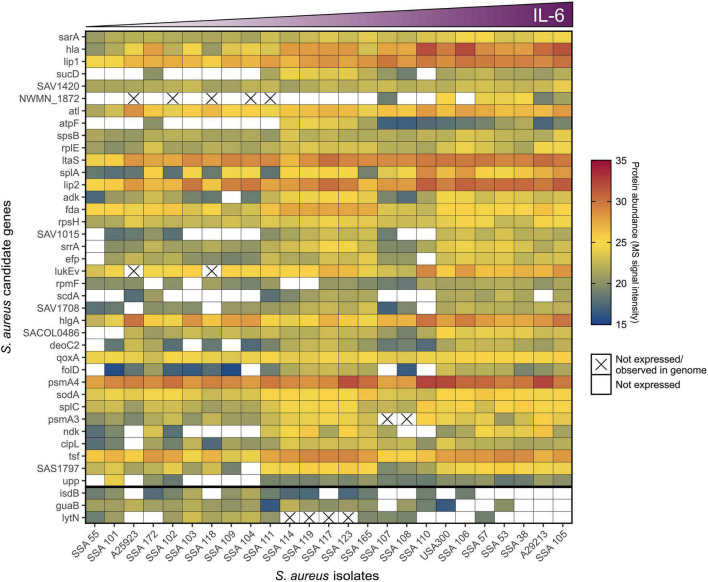
The pro-inflammatory potential of *S. aureus* is determined by differences in gene regulation. Genetic and proteomic characteristics of statistically significant candidates (Table 1) that positively (*n* = 37, ordered by significance top to bottom) or negatively (*n* = 3, separated to bottom of heat map) correlated to *S. aureus* secretomes’ ability to induce IL-6 in keratinocytes (ordered by level of IL-6 induction left to right). Protein abundance, i.e., MS signal intensity of candidates within the different *S. aureus* secretome samples, is indicated by color scale, where white is no recorded signal. The cross signifies the absence of a functional gene.

To address whether the observed phenotypic differences in pro-inflammatory potential of *S. aureus* clinical isolates was associated with defined differences in the bacterial genome, an overlay matrix (Figure 5) was generated to compare the presence or absence of specific genes with the relative abundance of the corresponding protein for the *S. aureus* candidates presented in Table 1 that were significantly associated with keratinocyte IL-6 induction. If *S. aureus* isolates with no or minimal effect on IL-6 lack the relevant gene for proteins positively correlated with IL-6 induction, this would indicate that the observed phenotypic differences are due to genetic variance. However, non-IL-6 inducing *S. aureus* supernatants showed some expression of bacterial proteins correlated with IL-6 expression (Figure 5), and even when bacterial protein expression was not detected, a functional gene copy was often present. Therefore, the observed heterogeneity in induction of IL-6 by *S. aureus* supernatants is more readily explained by a variation in bacterial protein expression, post-translational modifications, and/or targeted protein degradation or stabilization, rather than the presence or absence of a specific bacterial gene.

## Discussion

Keratinocyte cancer and its precursor stages are associated with an increased abundance of *Staphylococcus aureus* (Kullander et al., 2009; Wood et al., 2018; Andersson et al., 2019; Madhusudhan et al., 2020). *S. aureus* can secrete products which induce pro-inflammatory signaling in the skin (Otto, 2014; Liu et al., 2017, 2018), and chronic inflammation is linked to skin cancer development and progression (Mueller, 2006; Grivennikov et al., 2010). It therefore seems plausible that *S. aureus* colonizing pre-malignant lesions may chronically activate pro-inflammatory signaling pathways that could aid cancer progression. However, *S. aureus* has considerable strain-level heterogeneity (Ziebandt et al., 2010; Bonar et al., 2016), and not all strains may adversely impact the inflammatory state of the skin. The phenotypes of skin lesion-associated *S. aureus* in relation to cutaneous inflammation have previously not been reported. Here, we demonstrate that secretome components of *S. aureus* isolated from AK, IEC, and SCC lesions can induce cancer-linked pro-inflammatory responses in cultured human keratinocytes and murine skin.

The pro-inflammatory cytokines IL-6, IL-8, and TNFα possess cancer-promoting functions and are commonly overexpressed in many malignancies including keratinocyte cancers (Gambichler et al., 2006; Lin and Karin, 2007; Lederle et al., 2011; Moussai et al., 2011; Ghahartars et al., 2021). In line with this, we show these cytokines are overexpressed in skin biopsies of AK, IEC, and SCC. We demonstrate that secretomes from *S. aureus* isolated from photo-damaged skin, AK, and SCC can cause enhanced gene expression of IL-6, IL-8, and TNFα in human keratinocytes. Some secretomes were particularly potent at inducing these pro-tumorigenic inflammatory mediators, and hence, colonization with certain *S. aureus* strains may more readily contribute to a tumor-promoting microenvironment in the skin than others. Further, we show that keratinocytes challenged with IEC or SCC-associated *S. aureus* secretome secrete significantly increased levels of IL-6 protein. Interestingly, the overexpression of IL-6 in cultured human keratinocytes has previously been found to result in enhanced angiogenesis, proliferation and migration, and resistance to apoptosis (Jee et al., 2001; Lederle et al., 2011). Type 1 cytokine signaling through IL-6 typically propagates systemic pro-inflammatory responses (Turner et al., 2014). Consistent with this, *S. aureus* secretomes identified to be strong IL-6 inducers *in vitro* also triggered pronounced innate immune responses in murine skin. Thus, skin colonization with IL-6-inducing *S. aureus* may cause a persistent accumulation of lingering immune cells, which has been linked to cancer progression (Grivennikov et al., 2010).

In both our *in vitro* and *in vivo* experiments, there were clear strain-level differences in the potency of *S. aureus* to induce pro-inflammatory skin responses. Proteomic and genetic analyses revealed that the observed difference in *S. aureus* phenotypes was due to variances in regulation and secretion of potent cytolytic toxins, such as leucocidins, hemolysins, and PSMs, as well as multiple serine proteases. The pro-inflammatory properties of these factors have been well-documented in the past (Zdzalik et al., 2012; Otto, 2014; Liu et al., 2017; Stentzel et al., 2017). PSM α3 and α4 were significantly correlated with IL-6 induction in keratinocytes, consistent with a recent study showing α-type PSMs to be potent inducers of a range of pro-inflammatory cytokines, including IL-6, in human primary keratinocytes and skin explants (Damour et al., 2021). PSMα peptides are known to mediate the release of membrane-embedded *S. aureus* lipoproteins (Hanzelmann et al., 2016), and lipoproteins trigger TLR2-dependent immune activation (Stoll et al., 2005; Nguyen and Götz, 2016; Mohammad et al., 2021). Interestingly, we identified a to-date uncharacterized lipoprotein (SACOL0486) associated with pro-inflammatory skin responses, alongside a number of other uncharacterized proteins that could play as-yet-unknown roles in the virulence of this important pathogen. We also found that lipoteichoic acid synthase was positively correlated with the pro-inflammatory *S. aureus* phenotype. Lipoteichoic acid is a separable cell wall-anchored amphiphilic glycolipid with pro-inflammatory and pathogenic properties that, of note, has been shown to encourage enhanced proliferation of adeno- and squamous cell carcinoma cells (Hattar et al., 2017). Lastly, further experimental evidence supports the results of our correlation analysis as the antibody neutralization of α-toxin, one of our identified candidates most strongly associated with IL-6 activity, in the *S. aureus* supernatants caused a significant reduction in IL-6-inducing potency. This is consistent with findings from previous studies that support α-toxins’ direct, and indirect, contributions to IL-6 induction in keratinocytes and a range of other cell types *in vitro* as well as *in vivo* mouse models (Onogawa, 2002, 2005; Hruz et al., 2009; Hong et al., 2014).

This study provides valuable insights into the secreted proteome of *S. aureus* isolates recovered from photo-damaged skin and different SCC developmental stages, and shows that strain-specific pro-inflammatory effects are most likely explained by differential regulation of toxins. A caveat of our study is that filter-sterilized culture supernatant from *S. aureus* not only contains secreted proteins but also intracellular proteins released by the lysis and turnover of bacterial cells, as well as other *S. aureus*-derived metabolites and molecules, such as DNA and RNA, that could promote immune activation. Moreover, the growth of *S. aureus* in liquid culture may produce different secreted products and/or at different quantities than during skin colonization due to higher nutrient availability, less environmental stress, and lacking interactions with other microbial species and host cells. Therefore, *in vivo* studies are required to establish whether *S. aureus* skin colonization indeed mediates lasting pro-inflammatory effects that can aid SCC development and progression. This could have important implications in the treatment of AK and prevention of IEC and SCC.

## Materials and Methods

### RNA Sequencing of Skin Cancer Biopsies

RNA sequencing was performed on four healthy skin samples and nine AK, six IEC, and five SCC lesion biopsies. Part of the biopsy was sectioned to confirm diagnosis *via* histology. The 24 skin samples were collected from 15 individuals at the Dermatology department in Princess Alexandra Hospital. Ethical approval was obtained by Metro South Human Research Ethics Committee and the University of Queensland Human Research Ethics Committee (HREC-11-QPAH-236, HREC-11-QPAH-477, HREC-12-QPAH-217, and HREC-12-QPAH-25). Written, informed consent was obtained from all patients prior to participation. Fresh biopsy tissues were immersed in RNA later (Life Technologies, Carlsbad, CA) and stored at −80°C until processing. RNA isolation was performed using the QIAGEN RNeasy Plus Mini kit. RNA concentration was measured on the Qubit fluorometer (Life Technologies, Carlsbad, CA) and RNA integrity determined using the 2100 Bioanalyzer (Agilent Technologies, Palo Alto, CA) on RNA Pico chips. RIN > 6 was set as minimum acceptable quality to proceed. RNA-Seq libraries of poly (A) RNA from 500 ng total RNA were generated using the TruSeq unstranded mRNA library prep KIT for AK, IEC, and SCC samples. TruSeq stranded mRNA library preparation kit was used to generate poly (A) RNA libraries from RNA extracted from normal skin. Libraries were sequenced (100 bp, paired-end) on the Illumina HiSeq 2500 platform (Illumina, San Diego, CA, United States). Quality of raw data was assessed using FastQC (version 0.11.2) (Andrews, 2010). Adapters and poor-quality sequences (∼6% of reads) were removed using Trim Galore v0.3.7 (Krueger, 2015). Trimmed reads were then aligned using the unstranded protocol in TopHat (Trapnell et al., 2012) against the Human Genome (hg19 build) guided with a human transcriptome generated from the GENCODE Gene annotation v17 (Harrow et al., 2012). Raw counts were normalized using TMM method using Bioconductor package edgeR version 3.3. Quantification of gene expression was based on counting the overlaps of mapped reads with genes annotated in the GENCODE gene annotation v17 and was conducted *via* HTSeq (Anders et al., 2015).

### Clinical Sampling of Skin Cancer and Precancerous Lesions

Individuals with AK, IEC, and/or SCC lesions were recruited at the Princess Alexandra hospital (Brisbane, Australia) under approved protocols HREC/16/QPAH/364 and HREC/11/QPAH/477. Exclusion criteria were the presence of chronic skin disorders, as well as current or recent use of antibiotics and/or topical agents to treat AK. Skin swabs from AK were collected from the forearm only, whereas those from IEC and invasive SCC were collected from any UV-exposed body site. Non-malignant control swabs were collected adjacent to each sampled lesion on an anatomically similar skin site. Sterile swabs were first dipped into 0.15 M sodium chloride solution, firmly rotated over the skin sampling area for 30 s, and then collected in sterile glycerol-saline solution and stored immediately at −80°C. Suspected IEC and SCC lesions were excised after swabbing and the diagnosis confirmed *via* histopathology.

### Isolation of *Staphylococcus* Clinical Strains

Swab samples preserved in glycerol were plated onto *Staphylococcus* genus-selective mannitol salt agar (MSA). After 48 h at 36°C, a representative sample of colonies from each MSA-positive plate were picked and restreaked onto new MSA plates. Golden colonies that formed a yellow halo on the red MSA agar were selected as indicative of the *S. aureus* species. After 24 h of growth, a single colony was transferred into tryptic soy broth and grown overnight. The next morning, a subsample of the live bacterial suspension was stored in 100% glycerol (1:1) and the remaining biomass was collected by centrifugation and resuspended in lysis buffer (500 mM NaCl, 50 mM TRIS-HCl [pH 8.0], 50 mM EDTA, 4% Sodium Dodecyl Sulfate) for DNA extraction and sequencing. Both the live glycerol stock and lysed bacteria were stored at −80°C.

### Species Identification

The MSA-positive isolates were first subjected to Gram staining, and Gram positive cocci were chosen for coagulase and catalase test following a standard protocol (Moyes et al., 2009; Reiner, 2010). For coagulase and catalase-positive isolates, a PCR was carried out using *S. aureus*-specific primers targeting the *nuc* gene as previously published (Kullander et al., 2009; Reuschenbach et al., 2011). A thermal extraction method was used to collect DNA from liquid cultures of the chosen isolates. Overnight cultures were pelleted by centrifugation and resuspended in 200 μL of ultrapure water. The cell suspension was then incubated in a heating block at 100°C for 10 min. Lysates were immediately transferred onto ice and centrifuged at 10,000 × g for 3 min. In addition, 2 μL of the supernatant were used in a 15 μL PCR assay using Taq DNA polymerase (0.1 μL per sample) with standard Taq buffer and 20 μm of each *nuc* primer (*nuc* forward 5′-GCGATTGATGGTGATACGGTT-3′; *nuc* reverse 5′-AGCCAAGCCTTGACGAACTAAAGC-3′). PCR cycle setting were as follows: 94°C for 2 min, followed by 37 cycles of 94°C for 1 min, 55°C for 30 s and 72°C for 1.5 min, and lastly 72°C for 3.5 min followed by a 4°C hold. The 263-bp PCR product was confirmed *via* visualization on a 1.5% agarose gel. Lastly, *nuc*-positive isolates were confirmed as *S. aureus via* 16S rRNA gene sequencing. To purify DNA for sequencing, the frozen vial of bacteria resuspended in lysis buffer was thawed and DNA extracted using a repeated bead beating step for cell lysis, followed by DNA purification utilizing the Maxwell^®^ 16 MDx instrument (Promega). The 16S rRNA gene was amplified using 27F and 1492R primers (27F 5′-AGAGTTTGATCCTGGCTCAG-3′; 1492R 5′-GGTTACCTTGTTACGACTT-3′), and subsequently the reaction mixture was purified and cleaned from unincorporated dyes, nucleotides, salts, and contaminants using the Agencourt^®^ CleanSEQ^®^ kit according to standard protocol. The PCR product was subjected to Sanger sequencing and the resulting amplicon sequence was used to query the GenBank database. Isolates that were Gram-positive cocci, catalase and coagulase-positive, were *nuc* PCR-positive, and confirmed affiliation with *S. aureus via* 16S RNA gene sequencing were included in this study and selected for secretome production.

### Whole Genome Sequencing of *Staphylococcus aureus* Isolates

The genomic DNA prepared from 34 of our *S. aureus* isolates was used to produce draft genome assemblies. DNA libraries were prepared with a Nextera Flex library preparation kit (Illumina, #20018705). Library preparation was run on the Mantis Liquid Handler (Formulatrix). Resulting amplified libraries were cleaned-up as per the “Clean Up Libraries” section in the manufacturer’s protocol. Each library was then quantified using the Quant-iT™ dsDNA HS Assay Kit (Invitrogen) and quality assessed *via* Agilent D1000 HS tapes (#5067-5582) on the TapeStation 4200 (Agilent # G2991AA) as per the manufacturer’s protocol. Nextera DNA Flex libraries were pooled at equimolar amounts of 0.5 nM per library to create a sequencing pool. The library pool was then again quantified in triplicates using the Qubit™ dsDNA HS Assay Kit (Invitrogen). Library quality control was performed using the Agilent D1000 HS tapes (#5067-5582) on the TapeStation 4200 (Agilent # G2991AA). The library was prepared for sequencing on the NextSeq500 (Illumina) using NextSeq 500/550 High Output v2 2 × 150bp paired end chemistry according to manufacturer’s protocol. Approximately 0.5 GB of sequence data was produced per sample. Raw reads for isolated strains were processed with Trimmomatic (ver. 0.39; LEADING:3, TRAILING:3, SLIDINGWINDOW:4:15, MINLEN:50) for quality filtering (Bolger et al., 2014). Quality filtered reads were then assembled using SPAdes (ver. 3.14.0) (Bankevich et al., 2012) as part of the Shovill assembly pipeline (ver. 1.1.0, T. Seeman, unpublished, https://github.com/tseemann/shovill “–isolate,” –minlen 1000), with the completeness and contamination of each assembly evaluated using CheckM (ver. 1.1.2) (Parks et al., 2015) and taxonomy assigned using GTDB-Tk (ver. 1.3) (Chaumeil et al., 2019).

A phylogenetic tree was constructed for the assembled 34 *S. aureus* draft genomes based on a core-alignment of single nucleotide polymorphisms (SNPs) following the removal of recombinant regions. Draft genomes were aligned against the *S. aureus* USA300_FPR3757 reference genome (RefSeq accession GCF_000013465.1) using Parsnp (ver. 1.5.6) (Treangen et al., 2014), with recombinant regions identified and removed using Gubbins (ver. 3.0.0) (Croucher et al., 2015). A maximum-likelihood phylogenetic tree was constructed using RAxML (ver. 8.2.12) using a general time-reversible nucleotide substitution model with gamma correction for site variation (GTRGAMMA) and 1,000 bootstraps. The phylogenetic tree was rooted on the reference USA300_FPR3757 genome and visualized using the ggtree package (ver. 2.2.4) (Yu et al., 2017) in R (ver. 4.0.2). Multi-locus sequence typing (MLST) was performed with MLST ver. 2.17.6, https://github.com/tseemann/mlst, which scans against PubMLST typing schemes^1^ (Jolley et al., 2018).

### Production of *Staphylococcus aureus* Sterile Culture Supernatant

For secretome production, *S. aureus* strains were cultured in chemically defined staphylococcal medium (DSM) (adapted from Hussain et al., 1991; composition in Supplementary Table 3) as this medium without added proteins or peptides is compatible with mass spectrometry. Clinical isolates were plated fresh from glycerol stock onto MSA and left to grow for 24 h at 37°C and 5% CO_2_. A single colony was then used to inoculate 6 ml DSM within 15 mL round bottom tubes and grown with shaking (200 rpm) at 37°C. After overnight growth, the optical density at 600 nm (OD 600) of the cultures was measured, and a volume of the culture was used to inoculate 100 mL DSM so as to provide an initial OD 600 of 0.15. The cultures were shaken at 200 rpm and incubated at 37°C for 24 h, with growth monitored *via* OD readings. After 24 h, the final OD 600 value was measured. Then, the cultures were centrifuged at 800 × g for 20 min at 4°C to pellet the cell biomass. The supernatant was carefully transferred into a fresh tube and centrifuged at 3,000 x g for 20 min at 4°C. The resulting supernatant was filter-sterilized through 0.2 μm regenerated cellulose (Corning; CLS431222) and partitioned into single-use aliquots, which were stored immediately at −80°C. Uninoculated DSM was treated precisely the same way to use in experiments as a negative control. The sterility of the secretome samples was routinely confirmed by plating aliquots of the filtered bacterial supernatant onto agar and monitored for growth at 37°C. For experiments, aliquots were freshly thawed, used immediately, and discarded after use.

Initially, sterile culture supernatant was produced from four of our *S. aureus* isolates in three biological replicates, i.e., three different colonies were inoculated, grown for 24 h, and sterile supernatant collected as described above. As the effect of the matching replicate supernatants on keratinocyte cytokine induction was highly consistent, there was only one biological replicate of supernatant produced and tested from the remaining isolates in our functional assays (methods below).

### Keratinocyte Cell Culture and Experiments

HaCaT cells were cultured in DMEM medium supplemented with 10% fetal calf serum (FCS) and 100 U/mL penicillin/streptomycin at 37°C and 5% CO_2_. HaCaT experiments were performed up to passage 30 in serum-free culture conditions using DermaLife K keratinocyte basal medium (Lifeline; SKU: LL-0007). Primary human keratinocytes were isolated from abdominal skin, foreskin, or actinic keratosis lesions collected at the Princess Alexandra Hospital Dermatology Centre (Brisbane, Australia) with patient consent and institutional approval (HREC/11/QPAH/477). Primary keratinocytes were cultured in DermaLife K keratinocyte basal medium supplemented with 10 μM Rho Kinase inhibitor Y-27632 (Sigma Aldrich; SCM075). Experiments on primary keratinocytes were performed up to passage eight. For *S. aureus* secretome stimulation assays, keratinocytes were seeded in 96-well plates and, unless otherwise stated, experiments were initiated at approximately 70% cell confluence. The keratinocytes were then incubated with sterile culture supernatant of *S. aureus* and bacterial medium control, diluted 1:40 in DermaLife medium, for 24 h. By end point, keratinocyte cultures reached confluence and cell supernatant was collected for cytokine quantification and the cell viability assessed as described below in 4.8 and 4.9, respectively.

### Assessment of Cytokine Protein Levels

To measure IL-6 in keratinocyte conditioned media after challenge with *S. aureus* secretome, the HEK-Blue™ IL-6 reporter cell line from InvivoGen was used according to the provided standard protocol. This cell system employs HEK293 cells stably transfected with the human IL-6R gene and STAT3-inducible reporter gene of secreted embryonic alkaline phosphatase (SEAP). The presence of IL-6 in keratinocyte supernatant triggers STAT3 activation in transfected HEK293 cells and subsequent production of SEAP. SEAP levels were monitored by QUANTI-Blue™ colorimetric assay at an absorbance of 650 nm. Statistical analyses were performed with GraphPad Prism version 8.3.1 for Windows (GraphPad Software, San Diego, CA), with *p* ≤ 0.05 considered statistically significant. To validate cytokine results, a multiplex bead array kit by BioLegend was used according to the provided manual (LEGENDplex™ Human Inflammation Panel 1 [13-plex] with V-bottom Plate; Cat#740809). The assay was performed on 25 μL of undiluted keratinocyte culture supernatant and results were acquired on the CytoFLEX flow cytometer platform (Beckman Coulter; Cat#B53000). Data was analyzed with BioLegend’s LEGENDplexTM Data Analysis Software. Standard was prepared in technical duplicates and samples in biological triplicates. The standard curves for each monitored marker had a CV ≤ 1.7%.

### MTT (3-(4,5-Dimethylthiazol-2-yl)-2,5-Diphenyltetrazolium Bromide) Assay

To assess keratinocyte cell activity, the MTT assay was employed as developed by Mossmann (1983) with MTT purchased by Invitrogen™ (M6494). Keratinocyte culture medium was replaced with phenol-free medium containing 0.5 mg/ml MTT solution. After 4-h incubation at 37°C, the purple formazan crystals were dissolved in 60 μL dimethyl sulfoxide. The plates were incubated at 37°C for 10 min, then shaken for 1 min on a plate shaker, and finally absorbance measured spectrophotometrically at 540 nm. Healthy, untreated cells served as control to normalize to (=100% activity) and cells that were killed with lysis solution (Promega, G1821) served as 0% activity control.

### RNA Extraction and Real-Time Reverse Transcription Quantitative PCR

Keratinocytes were cultured in a 12-well dish until 60–70% confluent followed by treatment with *S. aureus* secretome diluted 1:40 in keratinocyte medium. Cells were washed with PBS and collected after 6 h by dislodging cells directly in RLT lysis buffer (Qiagen, 79216). RNA extraction was performed using the RNeasy Mini Kit (Qiagen, 74106) as per given protocol. Samples were eluted in 50 μL of nuclease-free water (Ambion, AM9937). To remove potential DNA contamination, the TURBO DNA-free kit by Ambion (AM1907) was used according to the manufacturers user guide. RNA concentration was measured using a NanoDrop Lite spectrophotometer (Thermo Fisher Scientific, 0716). cDNA was synthesized from 1 μg of RNA using the SuperScript III First-Strand Synthesis System (Invitrogen, 18080051). Real-Time Reverse Transcription Quantitative PCRs (RT-qPCRs) were run on Quantstudio7 Flex real-time PCR system, 384-well plate reader using Sybr Green assay (Applied biosystems, 4309849). Primers were synthesized by Integrated DNA Technologies and sequences were as follows: IL-6 forward GGCACTGGCAGAAAACAACC and reverse CACCAGGCAAGTCTCCTCAT; IL-8 forward ACCACCGGAAGGAACCATCT and reverse GGCAAAACTGCACCTTCACAC; TNF-α forward GTTCCC CAGGGACCTCTCTC and reverse GAGGGTTTGCT ACAACATGGG. To account for variations in RNA yield and cDNA synthesis efficiency, TATA box-binding protein (TBP) was chosen as the housekeeping gene to normalize against (forward CCGGCTGTTTAACTTCGCTT and reverse CCAAGAAACAGTGATGCTGGGT). Fold change in gene expression was calculated *via* the ΔΔCt method. Keratinocytes treated with bacterial media DSM served as control. Additionally, a reverse transcriptase negative cDNA sample and a water control were included in each assay.

### Keratinocyte Infection Experiment

Single colonies of four *S. aureus* isolates were inoculated in DSM and growth curves monitored for 24 h. The cultures were then clarified by centrifugation at 800 × g for 20 min and the supernatant of each culture was collected and divided into two tubes. One aliquot was filter-sterilized through 0.2 μm regenerated cellulose (Corning; CLS431222) to remove bacteria but retain *S. aureus* secretome, and the other was left untreated to retain live bacterial cells. Both unfiltered and filtered supernatants were spread on agar plates for bacterial live cell counts and to confirm sterility, respectively. HaCaT keratinocytes, which were seeded in 96-well plates the day prior, were then exposed to either the sterile *S. aureus* supernatant diluted 1 in 10 in keratinocyte media, or the supernatant containing live *S. aureus* diluted 1 in 10 in keratinocyte media, corresponding to an MOI of 1. After 24-h co-incubation, the keratinocyte cell viability and IL-6 secretion was assessed *via* MTT assay and HEK-Blue™ IL-6 assay as described above, respectively. This experiment was carried out in three biological replicates, i.e., three independent cultures of *S. aureus*, each in at least two technical replicates of HaCaT cells.

### *In vivo* Experiments: Mouse Tissue Collection, Cell Isolation, Antibody Staining, and Flow Cytometry

Six- to eight-week-old female C57BL/6J mice were purchased from Animal Resources Centre (Perth, Australia). Mice were housed at the Translational Research Institute Biological Research Facility which is operated as Specific Pathogen Free. Animal experiments were approved by The University of Queensland Animal Ethics Committee in accordance with the National Health and Medical Research Council of Australia guidelines for care and use of laboratory animals (UQDI/TRI/153/17). After anesthetizing with isoflurane, mice ear pinnae were injected intradermally with 20 μL of sterile *S. aureus* secretome or the controls PBS or DSM. To ensure precise delivery of treatment into the dermal layer of ear pinnae, a 30-gauge needle (BRAUN Omnican 50 *Insulin Syringes* 30G × 5/16) was used. Intradermal injection was considered successful when the formation of a wheal was observed and a sample was excluded if the injected secretome leaked excessively.

Then, 24 h after intradermal injection with *S. aureus* secretome or control, mice were euthanized by CO_2_ asphyxiation and the ears were harvested. Mice ears were split into dorsal and ventral halves with fine curved forceps and placed with the dermis side down in 2.5 mg/mL Dispase II (Roche) in PBS for 45 min at 37°C in 5% CO_2_. The dermis and epidermis were then separated and placed in PBS containing 1 mg/mL collagenase D (Roche) and 0.2 mg/mL DNase I (Thermo Fisher Scientific). The tissue was further disrupted into small pieces using surgical scissors to aid with isolation of single cell suspension. Samples were incubated for 45 min at 37°C and filtered through a 70 μm nylon cell strainer and rinsed through with 10 mL PBS buffer. The cell suspension was centrifuged at 350 × g for 5 min at 4°C. The supernatant was discarded and cell pellets disrupted and transferred into fluorescence-activated cell sorting (FACS) tubes.

The cells were stained with 1:100 Fcγ receptor blocking anti-CD16/32 (2.4 G2; eBioscience) and 1:250 LIVE/DEAD Fixable Aqua Dead Cell Stain (Thermo Fisher Scientific) in PBS. After 20-min incubation at 4°C in the dark, cells were washed with FACS buffer (2% fetal bovine serum (FBS), 2 mM EDTA, PBS) and centrifuged at 350 × g for 5 min at 4°C. After discarding the supernatant, cell pellets were resuspended in 50 μL of FACS buffer containing the following fluorescently conjugated antibodies: APC-anti-F4/80 (clone BM8; AbNo 570; eBioscience 1:200); Percp Cy5.5-anti-CD45 (clone 30-F11; AbNo 719; Biolegend 1:200) 1; PE/Cy7-anti-CD11c (clone HL3; AbNo 262; BD; 1:200); APC/Cy7-anti-MHC Class II (clone M5/114.15.2; AbNo 482; Biolegend 1:400); PE-anti-EPCAM PE (clone G8.8; AbNo 328; Biolegend 1:200); PB-anti-CD11b (clone M1/70; AbNo 564; Biolegend; 1:200); AF594-anti-TCRβ (clone H57-597; AbNo 808; Biolegend 1:200); AF700-anti-Ly6G (clone 1A8; AbNo 717; Biolegend; 1:200); BV711-anti-Ly6C (clone HK1.4; AbNo 718; Biolegend 1:400). After a 30-min incubation at 4°C in the dark, the cells were washed twice with 500 μL FACS Buffer and then again centrifuged at 350 × g for 5 min at 4°C. The supernatant was discarded and samples gently vortexed to dissociate the cell pellet which was then resuspended in 250 μL FACS buffer before analysis on the BD LSRFortessa™ X-20 flow cytometer.

### Mass Spectrometry Analysis of *Staphylococcus aureus* Supernatant

Protein concentration of *S. aureus* secretome was determined *via* DirectDetect^®^ infrared spectrometer (Merck) as well as BCA assay according to manufacturer’s protocol. *S. aureus* secretomes were diluted to 1 μg protein per μL in 10 mM tris(2-carboxyethyl)phosphine (TCEP), 40 mM chloroacetamide, and 1% sodium deoxycholate in 100 mM Tris pH 8.5. An aliquot of 10 μg of protein was heated for 5 min at 95°C and diluted 1 in 10 in water, then 0.2 μg of sequencing grade trypsin (Promega) was added and incubated overnight at 37°C. The next day, trifluoroacetic acid was added to 0.5%, and the samples were centrifuged at 13,000 × g for 10 min. The supernatant collected and the peptides were cleaned-up using 20 μg capacity C-18 tips (Glygen). After rehydrating the membrane twice with buffer B (80% acetonitrile, 0.1% formic acid), and equilibrating in buffer A (0.1% formic acid) 3 times, the peptides was bound using 10 pipetting cycles. The tip was washed once with buffer A and then peptides were eluted in buffer B.

Peptides were analyzed on a Thermo U3000 nano-HPLC system coupled to a Thermo Q Exactive Plus Orbitrap mass spectrometer. The HPLC setup used a C-18 trap column (DX160454) and a 50 cm EasySpray C-18 analytical column (ES803A) from Thermo Fisher Scientific. Mobile phases were A: 0.1% formic acid, and B: 80% acetonitrile with 0.1% formic acid. A total of 4 μL of sample equivalent to 1 μg of peptides were loaded in 2% B, and peptides eluted over a gradient from 2 to 50% B over 45 min at 250 nL/min. An EasySpray source was ran in positive ion data-dependent analysis mode with settings typical of peptide analyses. Briefly, full MS scans were acquired at 70,000 resolution, automatic gain control target was 3e6, with a maximum injection time of 50 ms. MS2 fragmentation was carried out on the top 10 precursors, excluding 1+ precursors. Precursor isolation width was 1.4 m/z and normalized collision energy was 28. MS2 resolution was 17,500 with an automatic gain control target of 5e5. Maximum injection time was 50 ms and the exclusion window was 10 s.

MaxQuant (Tyanova et al., 2016a) was used for peptide identification against the reviewed UniProtKB database (SwissProt; *S. aureus;* 11.10.2018; 11,075 proteins) using the “match between runs” function, normalization by label-free quantification and refining to a minimum of one unique peptide. The MaxQuant outputs were imported into Perseus (Tyanova et al., 2016b) and after the removal of proteins only identified by site, reverse sequences, and contaminants, the remaining 348 proteins were subjected to further analyses. The LFQ normalized MS intensity values were log2 transformed and missing values were imputed from normal distribution.

To establish whether there was a relationship between *S. aureus* protein abundance and the previously established keratinocyte IL-6 response, a Spearman’s rank correlation analysis for each individual protein was performed using the rcorr() function in the Hmisc package for R v3.6.1. Raw *p*-values were corrected for multiple testing using the “fdr” method and considered significantly correlated if the FDR was below 5% (FDR < 0.05).

To determine the presence/absence of significantly IL-6 correlated genes within individual isolates, translated coding sequences for the 34 *S. aureus* genomes were predicted by Prodigal (ver. 2.6.4) (Hyatt et al., 2010) as part of Prokka (ver. 1.14.6) (Seemann, 2014). Translated proteins were then aligned with blast + (ver. 2.9.0; *e*-value = 0.00001) (Camacho et al., 2009) against the IL-6 correlated protein sequences, with a gene considered present when at least 70% of its sequence was aligned with 70% sequence identity.

### Alpha Toxin Experiments

Recombinant *S. aureus* α-toxin and mouse monoclonal antibody [8B7] against α-toxin N-terminal were purchased from abcam (ab233724 and ab190467). Polyclonal anti-Staphylococcal α-toxin antibody produced in rabbit, and normal rabbit serum as control, were obtained from Sigma-Aldrich (S7531 and R9133). For the α-toxin neutralization experiments, the monoclonal antibody was serial diluted in serum-free keratinocyte media and added to diluted *S. aureus* secretome. The polyclonal antiserum and rabbit serum control were added to diluted *S. aureus* secretome at a 1:200 dilution. Antibody was allowed to bind for 30 min at 37°C with occasional shaking. The mixture was then added to keratinocytes grown in 96-well plate and after 24 h, IL-6 measurement *via* HEKblue cell reporter assay and viability assessment *via* MTT was carried out as described previously.

In a separate experiment, recombinant α-toxin (100 or 400 ng/ml) was added to diluted *S. aureus* secretome that typically does not induce an IL-6 response in keratinocytes, as well as one IL-6-inducing secretome. The *S. aureus* secretomes ± recombinant α-toxin were added to HaCaT cells in a 96-well plate and after 24 h of coincubation, the secreted IL-6 levels were measured by HEKblue cell reporter assay.

## Data Availability Statement

The genome sequencing data of our *S. aureus* clinical isolates have been deposited in the NCBI Sequence Read Archive under the Bioproject ID PRJNA754839. The raw MS data, the database search results, as well as the filtered and normalized protein data have been deposited to the publicly accessible platform ProteomeXchange Consortium via the PRIDE partner repository Perez-Riverol et al. (2019) under the dataset identifier PXD028604.

## Ethics Statement

The studies involving human participants were reviewed and approved by the Metro South Human Research Ethics Committee. The patients/participants provided their written informed consent to participate in this study. The animal study was reviewed and approved by the University of Queensland Animal Ethics Committee.

## Author Contributions

IF, MM, HS, PH, MH, and AK conceived and designed the experiments. AK, SC, and ST performed the experiments. AK and JZ analyzed the data. JZ, RL, NL, ZT, and SL contributed materials and/or analysis tools. AK wrote the manuscript. IF, MM, HS, PH, MH, and JZ helped editing. All authors contributed to the article and approved the submitted version.

## Conflict of Interest

The authors declare that the research was conducted in the absence of any commercial or financial relationships that could be construed as a potential conflict of interest.

## Publisher’s Note

All claims expressed in this article are solely those of the authors and do not necessarily represent those of their affiliated organizations, or those of the publisher, the editors and the reviewers. Any product that may be evaluated in this article, or claim that may be made by its manufacturer, is not guaranteed or endorsed by the publisher.

## Footnotes

1
https://pubmlst.org/
