# Supplementary material for: Secreted Toxins From Staphylococcus aureus Strains Isolated From Keratinocyte Skin Cancers Mediate Pro-tumorigenic Inflammatory Responses in the Skin
Source: Front Microbiol. 2022 Jan 25;12:789042. doi: 10.3389/fmicb.2021.789042 (PMC8822148; doi:10.3389/fmicb.2021.789042)
Supplement: Supplementary file 1 [file Data_Sheet_1.PDF]

Supplementary Material

1 Supplementary Figures

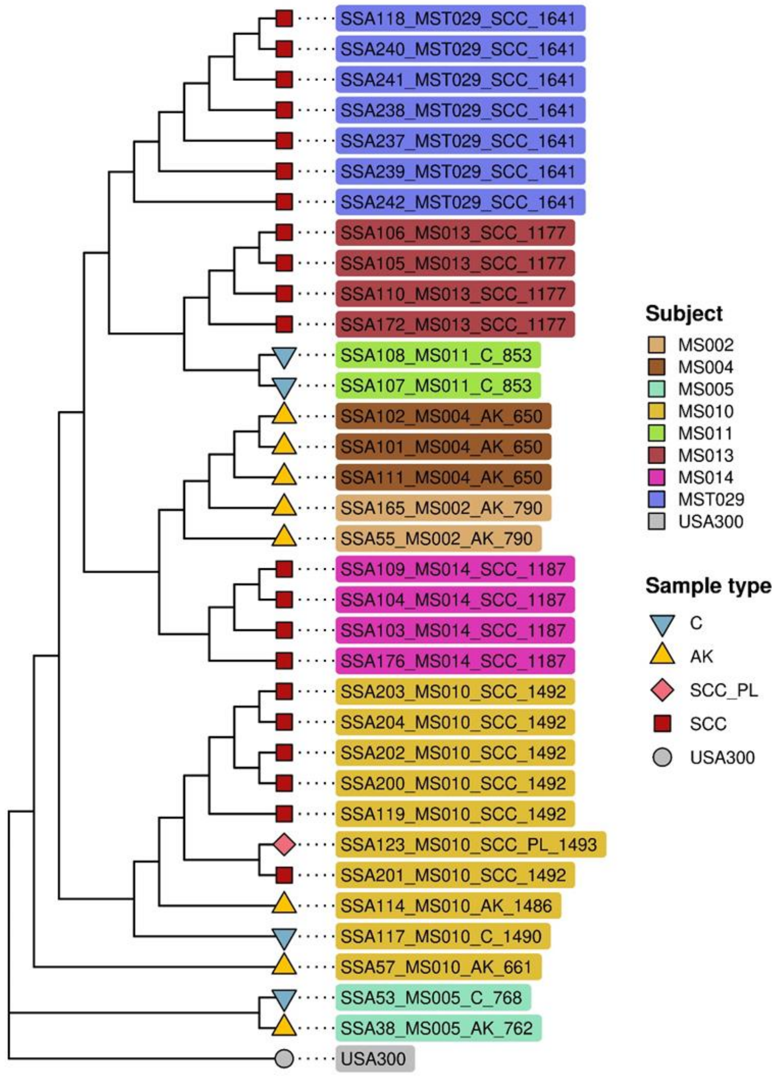

Supplementary Figure 1. ***S. aureus clinical isolates from the same subject are genetically alike, independent of the sample site.*** Phylogenetic tree of *S. aureus* clinical isolates originating from eight different subjects from photo-damaged non-malignant skin (C), actinic keratosis lesions (AK), squamous cell carcinoma (SCC) and SCC perilesional skin controls (SCC_PL), in reference to type strain USA300. Indicated is the IsolateID_SubjectID_Origin_Swab#.

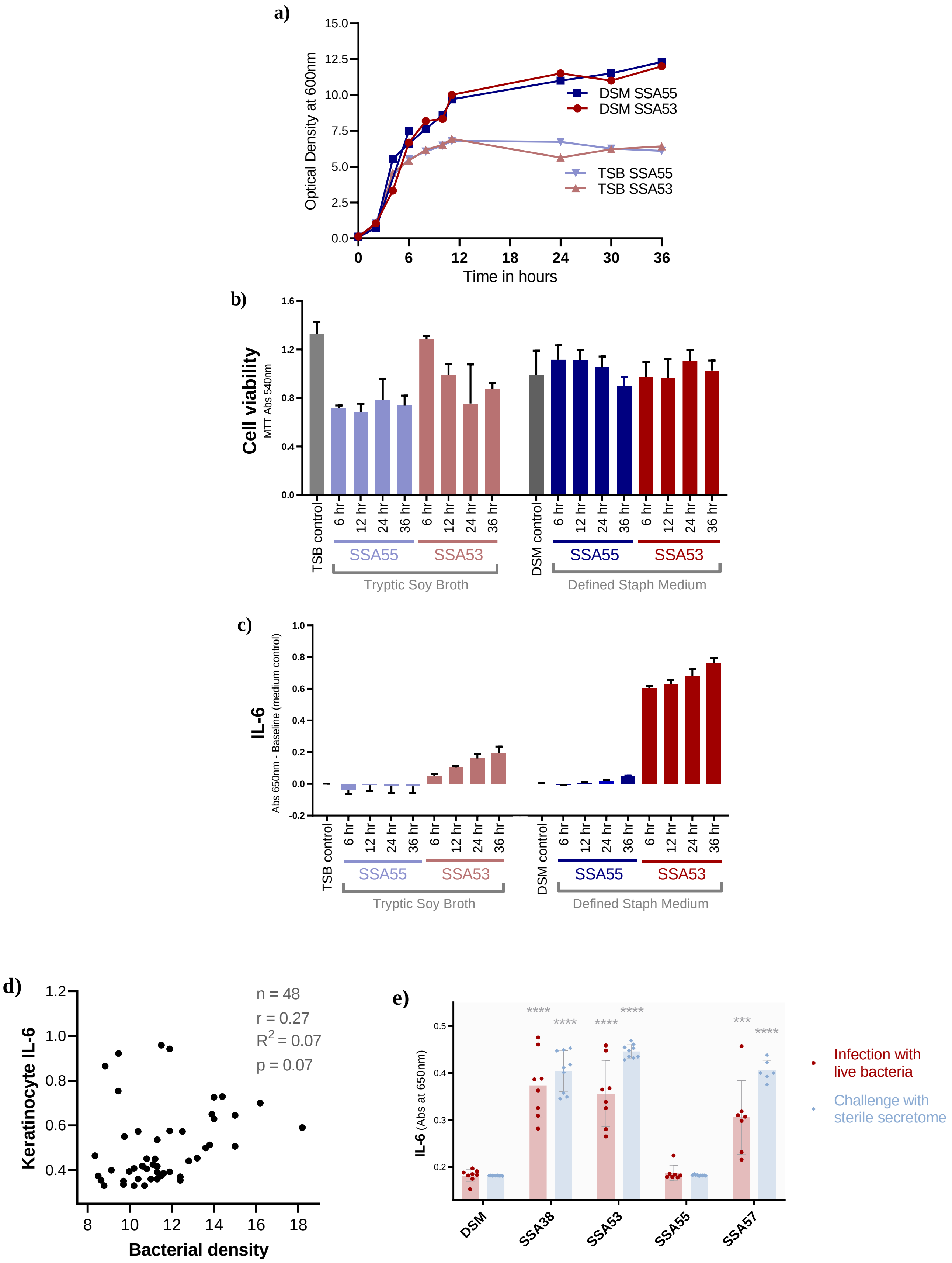
a)
Optical Density at 600nm
Time in hours
DSM SSA55
DSM SSA53
TSB SSA55
TSB SSA53
b)
Cell viability
MTT Abs 540nm
TSB control
6 hr
12 hr
24 hr
36 hr
SSA55
SSA53
Tryptic Soy Broth
DSM control
Defined Staph Medium
c)
IL-6
Abs 650nm - Baseline (medium control)
TSB control
SSA55
SSA53
Tryptic Soy Broth
DSM control
Defined Staph Medium
d)
n = 48
r = 0.27
R2 = 0.07
p = 0.07
Keratinocyte IL-6
Bacterial density
e)
IL-6 (Abs at 650nm)
DSM
SSA38
SSA53
SSA55
SSA57
Infection with live bacteria
Challenge with sterile secretome

Supplementary Figure 2. ***Keratinocyte IL-6 secretion in response to S. aureus secretome is independent of culture method, and similar as seen with infection with live S. aureus.*** **a)** Growth curves of *S. aureus* isolates SSA53 and SSA55 in tryptic soy broth (TSB) or defined staphylococcal medium (DSM). HaCaT keratinocyte **b)** cell viability and **c)** IL-6 levels measured after 24-hour exposure to *S. aureus* culture supernatant collected at 6, 12, 24 and 36 hours post inoculation of *S. aureus* isolates SSA53 and SSA55 grown in either TSB or DSM (mean +/- 1 SD; baseline = TSB or DSM control). Culture supernatant from *S. aureus* isolate SSA55 grown in TSB or DSM did not trigger IL-6 production in keratinocytes. On the other hand, SSA53 supernatant caused IL-6 induction in both media backgrounds, although the magnitude of the effect was higher in DSM, possibly due to increased levels of bacterial products in the supernatant due to improved growth (a) and lower toxicity to keratinocytes (b) **d)** Pearson's correlation between the optical density (600 nm) of *S. aureus* cultures at the time point of secretome collection (x-axis) and the IL-6 levels in keratinocyte conditioned media after exposure to the sterile secretome samples (y-axis) shows that the quantity of bacteria at the time point of secretome collection was not associated with its potency to induce IL-6 in keratinocytes. **e)** The level of IL-6 secreted by HaCaT keratinocytes in response to live bacteria and filter-sterilized culture supernatant (secretome) from *S. aureus* isolates (SSA38, 53, 55, and 57). Significance indicated compared to DSM control based on ordinary one-way ANOVA (p **** = <0.0001; p *** = <0.001).

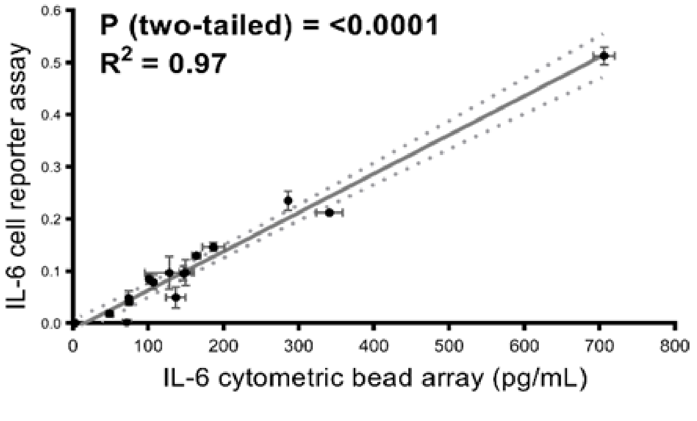

Supplementary Figure 3. ***IL-6 levels measured by cell reporter assay match results from cytometric bead array.*** Linear regression of IL-6 concentration in HaCaT keratinocyte conditioned media after treatment with 12 *S. aureus* secretomes as determined by cytometric bead array (x axis; mean of biological duplicates) and measured by HEKblue IL-6 reporter cell assay (y axis; mean of two biological replicates, three technical replicates each).

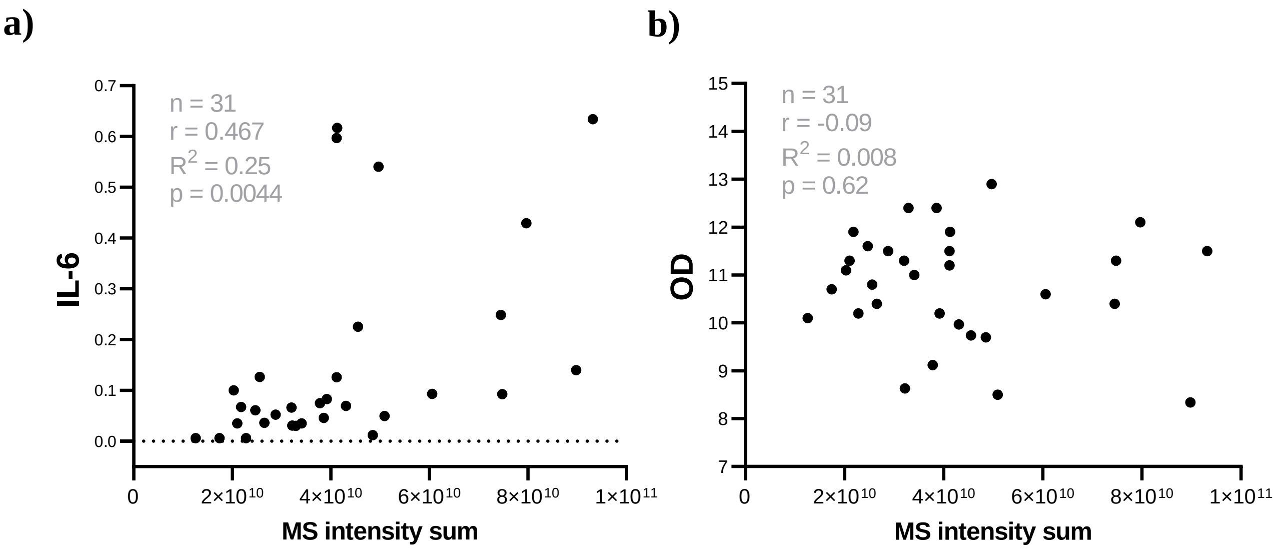

Supplementary Figure 4. ***The pro-inflammatory potential of S. aureus correlates to its level of overall protein secretion but is independent of bacterial cell density*****.** **a)** Significant correlation of induced IL-6 (cell reporter assay OD) from HaCaT cells by *S. aureus* secretomes with secretome total protein (sum of mass spectrometry intensities). **b)** Lack of correlation of bacterial cell density (OD 600 nm) at time of secretome harvest, with secretome total protein content.

2 Supplementary Tables

Supplementary Table 1. *Skin Staphylococcus aureus (SSA) isolate biobank*

Isolate ID	Subject ID	Sample site	Skin swab #	MLST
SSA 38	MS005	AK	762	8
SSA 53	MS005	C	768	8
SSA 55	MS002	AK	790	15
SSA 57	MS010	AK	661	1152
SSA 101	MS004	AK	650	15
SSA 102	MS004	AK	650	15
SSA 103	MS014	SCC	1187	1
SSA 104	MS014	SCC	1187	1
SSA 105	MS013	SCC	1177	72
SSA 106	MS013	SCC	1177	72
SSA 107	MS011	C	853	12
SSA 108	MS011	C	853	12
SSA 109	MS014	SCC	1187	1
SSA 110	MS013	SCC	1177	72
SSA 111	MS004	AK	650	15
SSA 114	MS010	AK	1486	97
SSA 117	MS010	C	1490	97
SSA 118	MST029	SCC	1641	508
SSA 119	MS010	SCC	1492	97
SSA 123	MS010	SCC_PL	1493	97
SSA 136	MS014	SCC_PL	1188	N/A
SSA 137	MS014	SCC_PL	1188	N/A
SSA 138	MS014	SCC_PL	1188	N/A
SSA 139	MS014	SCC_PL	1188	N/A
SSA 165	MS002	AK	790	N/A
SSA 166	MS009	C	853	N/A
SSA 167	MS009	C	853	N/A
SSA 168	MS009	C	853	N/A
SSA 169	MS009	C	853	N/A
SSA 170	MS013	SCC	1177	N/A
SSA 171	MS013	SCC	1177	N/A
SSA 172	MS013	SCC	1177	72
SSA 173	MS013	SCC	1177	N/A
SSA 174	MS013	SCC	1177	N/A
SSA 175	MS014	SCC	1187	N/A

SSA 176	MS014	SCC	1187	1
SSA 177	MS014	SCC	1187	N/A
SSA 178	MS014	SCC	1187	N/A
SSA 179	MS014	SCC	1187	N/A
SSA 200	MS010	SCC	1492	97
SSA 201	MS010	SCC	1492	97
SSA 202	MS010	SCC	1492	97
SSA 203	MS010	SCC	1492	97
SSA 204	MS010	SCC	1492	97
SSA 237	MST029	SCC	1641	508
SSA 238	MST029	SCC	1641	508
SSA 239	MST029	SCC	1641	508
SSA 240	MST029	SCC	1641	508
SSA 241	MST029	SCC	1641	508
SSA 242	MST029	SCC	1641	508
SSA 1673 - 7	B18	IEC	1673	N/A
SSA 1677 - 4	B18	IEC	1677	N/A
SSA 1681 - 42	B04	IEC	1681	N/A
SSA 1685 - 15	B04	IEC	1685	N/A
SSA 1691 - 45	B04	SCC	1691	N/A
SSA 1693 - 22	B04	SCC	1693	N/A
SSA 1701 - 12	F21	SCC	1701	N/A
SSA 1703 - 20	J11	SCC	1703	N/A
SSA 1705 - 10	J11	SCC	1705	N/A
SSA 1715 - 49	E20	SCC	1715	N/A
SSA 1717 - 6	E20	SCC	1717	N/A
SSA 1721 - 15	E20	IEC	1721	N/A
SSA 1723 - 5	E20	SCC	1723	N/A
SSA 1725 - 27	E15	SCC	1725	N/A
SSA 1727 - 7	E15	IEC	1727	N/A
SSA 1745 - 21	P04	IEC	1745	N/A
SSA 1751 - 11	D31	IEC	1751	N/A

C = non-malignant control skin
AK = actinic keratosis
IEC = intraepidermal carcinoma
SCC = squamous cell carcinoma
SCC_PL = SCC perilesional skin control
MLST = Multi-locus sequence typing

Supplementary Table 2. ***IL-6 inducing capacity of MS-characterized S. aureus secretomes.*** Proteins secreted by *S. aureus* which relative abundances (*i.e.* log2 transformed, LFQ normalised MS signal) significantly correlated to the secretomes' ability to induce IL-6 in keratinocytes.

***S. aureus* Isolate ID**	**Triplicate IL-6 measurements**			**Average IL-6**
**SSA 55**	0.298	0.357	0.337	**0.331**
**SSA 136**	0.312	0.349	0.333	**0.331**
**SSA 101**	0.318	0.328	0.348	**0.331**
**ATCC25923**	0.316	0.344	0.350	**0.337**
**SSA 139**	0.333	0.363	0.370	**0.355**
**SSA 172**	0.380	0.331	0.356	**0.356**
**SSA 137**	0.357	0.391	0.332	**0.360**
**SSA 138**	0.356	0.363	0.362	**0.360**
**SSA 102**	0.343	0.355	0.386	**0.361**
**SSA 103**	0.338	0.378	0.396	**0.371**
**SSA 118**	0.355	0.376	0.393	**0.375**
**SSA 109**	0.364	0.380	0.388	**0.377**
**SSA 176**	0.372	0.385	0.401	**0.386**
**SSA 104**	0.371	0.394	0.410	**0.391**
**SSA 111**	0.407	0.398	0.373	**0.393**
**SSA 167**	0.395	0.415	0.374	**0.394**
**SSA 114**	0.406	0.407	0.386	**0.400**
**SSA 119**	0.402	0.371	0.450	**0.408**
**SSA 117**	0.416	0.408	0.428	**0.417**
**SSA 123**	0.389	0.414	0.451	**0.418**
**SSA 165**	0.421	0.431	0.423	**0.425**
**SSA 107**	0.448	0.462	0.443	**0.451**
**SSA 108**	0.450	0.436	0.469	**0.452**
**SSA 110**	0.451	0.461	0.483	**0.465**
**USA300**	0.568	0.537	0.546	**0.551**
**SSA 106**	0.535	0.587	0.599	**0.574**
**SSA 57**	0.718	0.743	0.801	**0.754**
**SSA 53**	0.753	0.925	0.919	**0.866**
**SSA 38**	0.926	0.903	0.937	**0.922**
**ATCC29213**	0.869	0.955	1.002	**0.942**
**SSA 105**	0.941	0.985	0.951	**0.959**

Supplementary Table 3. ***Composition of defined staphylococcal medium***

Mixtures	Solute			Solvent		Solution			Notes	Vol	Substitute	
	Ingredient	g	mg	Solution	M	mg/ml	100x	200x			Ingredient	Conc.
Base	Sodium phosphate dibasic dehydrate ($Na_2HPO_4 \cdot 2H_2O$)	10								985.5	Na_2HPO_4-$7H_2O$	15.06g
	Potassium dihydrogen phosphate (KH_2PO_4)	3										
	CasAmino acids (acid hydrolysis of casein)	15										
	Magnesium sulfate heptahydrate ($MgSO_4 \cdot 7H_2O$)	0.5										
	Glucose ($C_6H_{12}O_6$)	10										
Vitamins	Biotin ($C_{10}H_{16}N_2O_3S$)		0.1	H_2O		2.2	1	2		10	NH_4OH (2 M)	50 mg/ml
	Nicotinic Acid ($C_6H_5NO_2$)		2	H_2O		15	20	40				
	D-Pantothenic Acid Ca salt ($C_9H_{16}CaNO_5^{\pm}$)		2	H_2O		50	20	40				
	Pyridoxal ($C_8H_9NO_3$)		4	HCl	1 N	50	40	80			Pyridoxal hydrochloride	
	Pyridoxamine dihydrochloride ($C_8H_{14}Cl_2N_2O_2$)		4	H_2O		500	40	80				
	Riboflavin ($C_{17}H_{20}N_4O_6$)		1	H_2O		1/3-15	10	20				
	Thiamin hydrochloride ($C_{12}H_{17}N_4OS^+$)		2	H_2O		50	20	40				
	Calcium chloride hexahydrate ($CaCl_2 \cdot 6H_2O$)		1	H_2O		70	10	20			$CaCl.2H_20$	
	Manganese sulfate ($MnSO_4$)		5	H_2O		219	50	100			MnSO4 x H_2O	
	Ammonium ferrous sulfate $(NH_4)_2SO_4FeSO_4 \cdot 6H_2O$		6	H_2O		256	5.5	11			Iron II Sulfate hexahydrate	
Amino Acids	Adenine sulphate ($C_{10}H_{12}N_{10}O_4S$)		20	HCl<br>Acetic Acid	1<br>1	4	200	400	Make 1 mL in each solvent and mix	2		
	Guanine Hydrochloride ($CH_5N_3 \cdot HCl$)		20	H2O		550	200	400	Made as slurry, then autoclaved	0.5		
	L-Tryptophan ($C_{11}H_{12}N_2O_2$)		100	HCl	0.5	50			filter sterilize	2		
